# Reversal of gene expression changes in the colorectal normal-adenoma pathway by NS398 selective COX2 inhibitor

**DOI:** 10.1038/sj.bjc.6605515

**Published:** 2010-01-19

**Authors:** O Galamb, S Spisák, F Sipos, K Tóth, N Solymosi, B Wichmann, T Krenács, G Valcz, Z Tulassay, B Molnár

**Affiliations:** 1 2nd Department of Medicine, Semmelweis University, Budapest, Hungary; 2 Hungarian Academy of Sciences, Molecular Medicine Research Unit, Budapest, Hungary; 3 1st Department of Pathology and Experimental Cancer Research, Semmelweis University, Budapest, Hungary

**Keywords:** NS398, cyclooxygenase-2, colorectal cancer, adenoma, whole-genomic microarray

## Abstract

**Background and aims:**

Treatment of colorectal adenomas with selective cyclooxygenase-2 inhibitors can contribute to the chemoprevention of colorectal cancer (CRC), but the molecular background of their effect is not fully understood. We analysed the gene expression modulatory effect of N-(2-cyclohexyloxy-4-nitrophenyl)-methanesulfonamide (NS398) on HT29 cells to be correlated with expression data gained from biopsy samples.

**Methods:**

HT29 colon adenocarcinoma cells were treated with NS398, and global mRNA expression was analysed on HGU133Plus2.0 microarrays. Discriminatory transcripts between normal and adenoma and between adenoma and CRC biopsy samples were identified using HGU133Plus2.0 microarrays. The results were validated using RT–PCR and immunohistochemistry.

**Results:**

Between normal and adenoma samples, 20 classifiers were identified, including overexpressed cadherin 3, KIAA1199, and downregulated peptide YY, glucagon, claudin 8. Seventeen of them changed in a reverse manner in HT29 cells under NS398 treatment, 14 (including upregulated claudin 8, peptide YY, and downregulated cadherin 3, KIAA1199) at a significance of *P*<0.05. Normal and CRC could be distinguished using 38 genes, the expression of 12 of them was changed in a reverse manner under NS398 treatment.

**Conclusion:**

NS398 has a reversal effect on the expression of several genes that altered in colorectal adenoma–carcinoma sequence. NS398 more efficiently inverted the expression changes seen in the normal-adenoma than in the normal-carcinoma transition.

Colorectal cancer (CRC) is one of the most frequent cancers in the world with a very high mortality rate even after surgical resection, radio- and chemotherapy (Cancer Research Campaign, 1999). It seems evident that CRC frequently follows and develops from adenomatous polyps (Leslie *et al*, 2002). Several studies suggest that non-steroidal anti-inflammatory drugs (NSAIDS) such as selective cyclooxygenase-2 (COX2) inhibitors have an anti-neoplastic effect (Giercksky, 2001; Cho *et al*, 2007; Saini *et al*, 2009).

Cyclooxygenase-2, one of the key enzymes of arachidone acid metabolism and prostaglandin synthesis, is described to be involved in the early stage of colorectal carcinogenesis. Cyclooxygenase-2 overexpression was shown in 85% of CRCs in proportion to normal tissue, and this expression alteration occurs in 50% of adenomas (Eberhart *et al*, 1994). Cyclooxygenase-2 can be activated through several cancer-associated biological pathways, such as Wnt- and Ras-related ones (Brown and DuBois, 2005). Pre-clinical studies suggest that the treatment of colorectal adenomas with selective COX2 inhibitors can contribute to the chemoprevention of CRC (Brown and DuBois, 2005).

The lack of COX2 expression characterises most normal tissues, but its level rapidly increases under mitogens and cytokines, which results in the accumulation of prostanoids (such as PGE2) in neoplastic and inflamed tissues (Eisinger *et al*, 2007). Elevated COX2 levels may lead to tumour development and expansion through activation of EGFR- and Tcf/Lef signal transduction pathways. Inhibition of apoptosis, interference with the immune system and facilitation of angiogenesis (vascular endothelial growth factor (VEGF) activation) and tumour invasion may also result from elevated PGE2 levels (Grösch *et al*, 2006).

Selective COX2 inhibitors seem to reduce the risk of developing colon cancer through COX2-dependent and -independent mechanisms. N-(2-cyclohexyloxy-4-nitrophenyl)-methanesulfonamide (NS398), a selective COX2 inhibitor, exerts its anti-carcinogenic effect by inducing apoptosis (Li *et al*, 2001; Nishikawa *et al*, 2004) and inhibiting cell cycle progression (Hung *et al*, 2000; Zhang and DuBois, 2001), angiogenesis (Abdelrahim and Safe, 2005; Huang *et al*, 2005) and metastasis (Abiru *et al*, 2002; Nishikawa *et al*, 2004; Yao *et al*, 2004; Chen *et al*, 2006; Banu *et al*, 2007; Leung *et al*, 2008). NS398 was reported to cause significant growth inhibition in HCA-7 colon carcinoma cells (Zhang and DuBois, 2001). It inhibits PGE2 synthesis and arrests cell cycle in G1 phase by enhancing p27KIP1 expression (Hung *et al*, 2000). NS398-dependent apoptosis in colon cancer cells occurs through a cytochrome *c*-dependent pathway (Li *et al*, 2001). Reducing VEGF levels with NS398 treatment refers to its anti-angiogenic effect (Abdelrahim and Safe, 2005; Huang *et al*, 2005). Inhibitory effects of NS398 on cancer invasiveness and metastatic growth have been proven both *in vitro* in cell culture (Abiru *et al*, 2002; Yao *et al*, 2004; Chen *et al*, 2006; Banu *et al*, 2007; Leung *et al*, 2008) and *in vivo* in animal model experiments (Chen *et al*, 2006; Leung *et al*, 2008). Therapeutic effects of NS398 can be exerted by downregulation of matrix metalloproteinase-2 expression (Yao *et al*, 2004; Leung *et al*, 2008), blocking of epidermal growth factor receptor transactivation (Banu *et al*, 2007) or inhibition of HGF-induced invasiveness (Abiru *et al*, 2002; Chen *et al*, 2006). However, the complete molecular background of NS398 treatment on colon adenocarcinoma cells has not been analysed yet.

The aims of this study were to analyse the gene expression modulating effect of NS398 selective COX2 inhibitor on the HT29 colon adenocarcinoma cell line and to correlate this effect to the modulation in gene expression observed during normal-adenoma and normal-CRC transition when biopsy samples were analysed.

## Materials and methods

### Cell culture

HT29 colon adenocarcinoma cells were cultured at 37°C with 5% CO_2_ in RPMI-1640 medium (Sigma-Aldrich, St Louis, MO, USA) containing gentamycin and 10% FCS. In six-well plates, 300 000 cells per well were cultured for 1 day, and were then treated with 10, 25 and 100 *μ*M NS398 (Sigma-Aldrich, diluted in DMSO) for 72 h in FCS-free medium. 0.1% DMSO was used as control. Total RNA was extracted from three samples treated with 100 *μ*M NS398 and from three untreated controls for microarray analysis. In parallel, 40 000 cells per slide were cytocentrifuged and fixed for immunocytochemical analysis.

### MTT cell proliferation assay

In 96-well plates, 5000 HT29 cells per well were maintained for 24 h in 100 *μ*l RPMI-1640 medium containing 10% FCS, after which, the cells were treated with 10, 25 and 100 *μ*M NS398 (Sigma-Aldrich, diluted in DMSO) for 48 or 72 h in FCS-free medium. A volume of 0.5 mg ml^−1^ of MTT (methylthiazolyldiphenyl-tetrazolium bromide, Sigma-Aldrich) was then added to each well, and the cells were incubated for 4 h at 37°C. The medium was carefully removed, and blue formazan – spawned from MTT by the mitochondrial dehydrogenase enzyme system of cells – was diluted in DMSO. Absorbance was measured at 570 nm using a Multiskan MS ELISA plate reader (Thermo Fisher Scientific Inc., Waltham, MA, USA).

### Laser microdissection

Samples were derived from surgically removed tissue from six patients with moderately differentiated, Dukes B stage, left-side CRC. In parallel, six adenoma specimens were collected. Paired control non-tumour tissues from patients were obtained from a clinically unaffected site near the resection end and were histologically normal. Tissue samples were immediately frozen in liquid nitrogen after surgery and were stored at −80°C until the cutting period. Frozen tissue was placed in a cryomold with Tissue Tek embedding medium on dry ice for 1 min. Frozen tissue specimens were cut in a series of 6-*μ*m-thick sections onto PALM membrane-mounted glass slides at −20°C. After cutting, the slides were taken into dry ice, and were stored at −80°C until microdissection for up to 48 h before staining and dissection. The frozen sections were fixed in ethanol series, and were stained using cresyl violet (Sigma-Aldrich). After staining the tissue, tumour and normal tissues were diagnosed by the pathologist. A total of 5000 epithelial cells were collected from each section using the PALM system (PALM, Bernried, Germany).

### Microarray analysis

Total RNA was extracted from HT29 cells using the RNeasy Mini Kit (Qiagen Inc., Germantown, MD, USA) and from LCM cells using the RNeasy Micro Kit (Qiagen Inc.), according to the manufacturer's instructions. The quantity and quality of isolated RNA were tested by measuring absorbance and capillary gel electrophoresis using the 2100Bioanalyzer and RNA 6000 Pico Kit (Agilent Inc., Santa Clara, CA, USA). Biotinylated cRNA probes were synthesised from 1 to 5 *μ*g total RNA and fragmented using the One-Cycle Target Labeling and Control Kit (http://www.affymetrix.com/support/downloads/manuals/ expression_s2_manual.pdf), according to the Affymetrix description. In case of LCM samples, two-cycle T7-based linear amplification was performed according to instructions of the manufacturer (Affymetrix Inc., Santa Clara, CA, USA). A volume of 10 *μ*g of each fragmented cRNA sample was hybridised into HGU133 Plus2.0 array (Affymetrix) at 45°C for 16 h. Slides were washed and stained using Fluidics Station 450 and an antibody amplification staining method according to the manufacturer's instructions. Fluorescent signals were detected by a GeneChip Scanner 3000 (Affymetrix). Fifty-three microarrays from colonic biopsy samples (11 normal, 20 villous adenoma, 22 CRC) had been hybridised earlier, their data files were used in a previously published study using different comparisons (Galamb *et al*, 2008a, 2008b, 2009) and are available in the Gene Expession Omnibus database (series accession numbers: GSE4183 and GSE10714).

### Statistical evaluation of mRNA expression profiles

#### Pre-processing and quality control

Quality control analyses were performed according to the suggestions of The Tumour Analysis Best Practices Working Group (Tumor Analysis Best Practices Working Group, 2004). Scanned images were inspected for artifacts; the percentage of present calls (>25%) and control of RNA degradation were evaluated. On the basis of evaluation criteria, all biopsy and HT29 measurements fulfilled the minimal quality requirements. In case of HT29 experiments, the similarity of the 3-3 biological replicates was stated using the Euclidean distance method (Supplementary Figure 1). Affymetrix expression arrays were pre-processed by gcRMA with quantile normalisation and median polish summarisation. Data sets are available in the Gene Expression Omnibus databank for further analysis (http://www.ncbi.nlm.nih.gov/geo/), series accession numbers: GSE15799, GSE15960, GSE4183 and GSE10714).

#### Further analyses

To identify differentially expressed features, significance analysis of microarrays (SAM) was used. The nearest shrunken centroid method (prediction analysis of microarrays=PAM) was applied for sample classification from gene expression data. Prediction analysis of microarrays uses soft thresholding to produce a shrunken centroid, which allows the selection of characteristic genes with high predictive potential (Tibshirani *et al*, 2002). Pre-processing, data mining and statistical steps were performed using R-environment with Bioconductor libraries. Annotation and functional classification of discriminatory genes were performed using the Affymetrix NetAffx system.

### Taqman RT–PCR

TaqMan real-time PCR was used to measure the expression of 12 selected genes using an Applied Biosystems Micro Fluidic Card System (Applied Biosystems, Foster City, CA, USA). The selected genes belonged to the PAM discriminatory genes between CRC and normal, and between adenoma and normal samples, and validated Taqman assays were available. The following commercially available Taqman Gene Expression Assays (Applied Biosystems) were applied: ABCA8 (Hs00200350_m1), TRPM6 (Hs00214306_m1), VWF (Hs00169795_m1), IL8 (Hs00174103_m1), LCN2 (Hs00194353_m1), CXCL1 (Hs00236937_m1), COL4A1 (Hs00266237_m1), MCAM (Hs00174838_m1), IL1RN (Hs00277299_m1), CXCL2 (Hs00236966_m1), DUOX2 (Hs00204187_m1) and SPP1 (Hs00167093_m1). Ribosomal RNA 18S (Hs99999901_s1) was used as reference. Using the Taqman Reverse Transcription Kit, 400 ng per sample of total RNA was reverse transcribed (Applied Biosystems). The quality of cDNA samples was checked by CK20/PBGD real-time PCR (F. Hoffmann-La Roche Ltd., Basel, Switzerland). Expression analysis of the selected genes was performed from 100 ng per sample cDNA template, using Taqman Low-Density Array for Gene Expression: Format 96a and Taqman Universal PCR Master Mix (Applied Biosystems). Measurements were determined using an ABI PRISM 7900HT Sequence Detection System as described in the products User Guide (http://www.appliedbiosystems.com). After enzyme activation at 95°C for 10 min, 40 PCR cycles were carried out (denaturation at 95°C for 15 s, annealing and extension at 60°C for 1 min). Data analysis was carried out using SDS 2.2 software, as described earlier (Galamb *et al*, 2008a). Baseline calculation and CT determination by individual thresholds according to the exponential phase of individual PCR reactions were automatically performed by the software. Variation of CT values of the three technical replicates was evaluated and accepted if it was under 0.5 cycle. Assays with an ΔRn value (difference between normalised reporter emission (Rn) of the sample template reaction and Rn of an unreacted sample) significantly differing from the average ΔRn should be excluded from further analysis. Relative quantification of gene expression was performed and fold change values were calculated using the ΔΔCT method (Livak and Schmittgen, 2001). The threshold cycle (CT) of the 18S ribosomal RNA endogenous control was used to normalise target gene expression (ΔCT) to correct for experimental variation. The extracted ΔCT values were grouped according to histological groups. Thereafter, Student's *t*-test was conducted to compare the expression values between groups.

### HT29 immunocytochemistry

For immunocytochemical analysis, 40 000 HT29 cells per slide were cytocentrifuged and fixed in aceton for 5 min, dried for 30 min at room temperature and stored at −20°C until staining. HT29 cells were immunolabelled using an anti-COX2 antibody and Novolink Polymer Detection System (Novocastra Laboratories Ltd., Newcastle upon Tyne, UK). Endogen peroxidase activity was neutralised by incubation for 30 min at room temperature in 0.5% hydrogen peroxide (in methanol). To reduce potential non-specific background, slides were treated with Protein Block reagent (Novocastra Laboratories Ltd.) for 5 min. After washing them twice in tris buffered saline for 5 min, the slides were incubated with rabbit monoclonal anti-human COX2 IgG (1:100, clone SP21, Thermo Fisher Scientific) for 1 h at room temperature. Antibodies were detected using the Novolink Polymer Detection System, followed by diaminodbenzidine substrate/chromogen (Novocastra). Haematoxylin co-staining was performed. The immunostained slides were digitalised using high-resolution MIRAX DESK instrument (Zeiss, Gottingen, Germany), and analysed with MIRAX Viewer version 1.11.43.0 and HistoQuant software (Zeiss). Total and COX2-positive cells (total cell number: approximately 1000) with × 32 magnification were counted in each sample. For statistical analysis, the *t*-test was performed to evaluate the difference of COX2-positive/total cell number ratios between NS398-treated and untreated control cells.

### Western blot analysis

HT29 colon adenocarcinoma cells were cultured for 1 day at 37°C with 5% CO_2_ in RPMI-1640 medium (Sigma-Aldrich) containing gentamycin and 10% FCS, and then treated with 25, 50 and 100 *μ*M NS398 (Sigma-Aldrich, diluted in DMSO) for 72 or 96 h in FCS-free medium. 0.1% DMSO was used as control. Soluble protein fractions were prepared from 1.5 × 10^6^ Triton X-100-treated cells in the presence of protease and phosphatase inhibitors, as described (Alpert *et al*, 2006). Protein samples (25 *μ*g) were electrophoresed (10% SDS–PAGE). Western blot analysis of COX2 (rabbit anti-human polyclonal COX2 antibody, Code: RB-9072, 1 *μ*g ml^−1^, Thermo Fisher Scientific) was performed as previously described (Tátrai *et al*, 2006). The ECL (Enhanced Chemiluminescent) technique (Dako, Glostrup, Denmark) and the Kodak Image Station 4000 MM instrument equipped with Molecular Imaging Software version 4.0 (Carestream Health Inc., Rochester, NY, USA) were used for visualisation and data evaluation.

### Ethical consideration

All routine colonic biopsy and surgical tissue specimens from patients were taken after informed consent and ethical permission was obtained for participation in the study.

## Results

### Colorectal adenoma and cancer-related mRNA expression patterns

Using PAM, between adenoma and normal biopsy samples, 20 classifiers were identified, including overexpressed cadherin 3, KIAA1199, forkhead box Q1 and downregulated carbonic anhydrase 7, glucagon, somatostatin, Spi-B transcription factor, claudin 8, bestrophin 4, peptide YY (sensitivity: 100%, specificity: 100%) (Table 1). Normal and CRC biopsy samples could be distinguished using 38 discriminatory genes (sensitivity: 90.91%, specificity: 100%) (Table 1). In LCM experiments, 65% of adenoma-related gene expression changes originated from epithelial cells, whereas 53% of CRC-related markers were epithelium derived.

### Validation of adenoma- and CRC-specific markers

All 12 measured genes showed a similar expression tendency than when detected by microarray analysis, and 9 of them correlated with the results obtained using Affymetrix microarrays at a significance of *P*<0.05. The expression changes of the selected genes are summarised in Table 2.

### Effects of NS398 treatment on gene expression in HT29 cells

**Figure 1:**
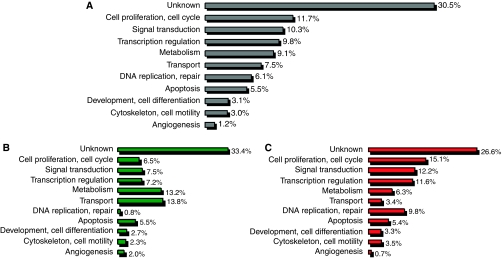
Functional classification of differentially expressed genes in HT29 cells under NS398 treatment. (**A**) Distribution of differentially expressed transcripts in the main cell functional groups. (**B**) Distribution of downregulated transcripts in the main cell functional groups. (**C**) Distribution of upregulated transcripts in the main cell functional groups.

In all, 1925 differentially expressed genes were identified between the NS398-treated and untreated control group using SAM at a significance of *P*<0.05 (Supplementary Table 1). A further feature selection criterion was the logFC (log fold change) value. Within the differentially expressed genes, 1156 at least two-fold-overexpressed genes were found with a logFC value higher than 1, whereas 769 at least two-fold downregulated genes were determined with a logFC value lower than −1. The expression of genes involved in cell proliferation and cell cycle regulation (such as overexpressed CDKN3, BTG2, TGFB1, CNOT8, KAT2B, RARRES3, CDKN2C, RARRES1, MAGED1, PPAP2A, MXD4, TENC1, SESN1, and downregulated CDCA4, VEGFA), intracellular signal transduction, transcription regulation, metabolic and transport processes and apoptosis (overexpressed CDKN2C, BIK, CASP6, TIA, DAPK3, and dowregulated ANXA1, CEBPB, CBX4) are mainly changed under NS398 treatment. However, the function of several differentially expressed transcripts is not known yet. The functional classification of genes is represented in Figure 1.

In correlation with the mRNA expression findings, significant dose-dependent cell proliferation inhibition was measured using MTT assay, which was carried out to optimise the treatment concentration of NS398 COX2 inhibitor.

### Changes in colorectal adenoma and cancer-related mRNA expression patterns under NS398 treatment

**Figure 2:**
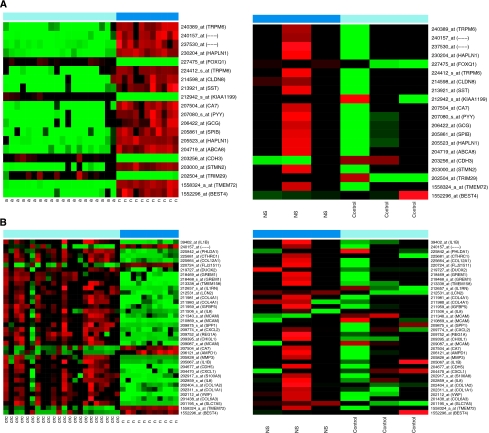
Changes in colorectal adenoma and cancer-related mRNA expression patterns under NS398 treatment. (**A**) Expression of adenoma *vs* normal discriminatory genes in biopsy samples and in HT29 colon adenocarcinoma cells under NS398 treatment (**B**). Expression of CRC *vs* normal discriminatory genes in biopsy samples and in HT29 colon adenocarcinoma cells under NS398 treatment.

Seventeen of these 20 genes changed in a reverse manner in HT29 colon adenocarcinoma cells under NS398 COX2 inhibitor treatment, 14 of them (including upregulated somatostatin, claudin 8, peptide YY, and downregulated cadherin 3, KIAA1199) at a significance of *P*<0.05 (Figure 2A). The expression of 12 of the 38 CRC-related markers (such as carbonic anhydrase 7, interleukin 8, melanoma cell adhesion molecule) was changed in a reverse manner under NS398 treatment (Figure 2B).

### HT29 immunocytochemistry and western blot results

**Figure 3:**
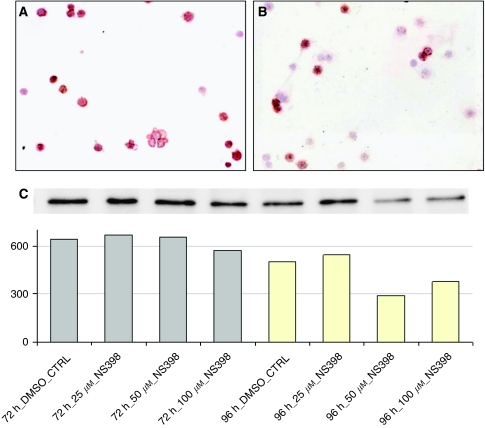
The decrease in COX2 protein expression under NS398 treatment. Dose-dependent inhibition of COX2 protein expression was observed under NS398 treatment. Strong granular and/or diffuse cytoplasmatic immunostaining was detected in COX2-positive cells. (**A**) COX2 protein expression in untreated control HT29 cells. (**B**) COX2 protein expression in HT29 cells treated with 100 *μ*M NS398 ( × 10 magnification, haematoxylin co-staining). (**C**) COX2 protein expression detected by western blot analysis. The diagram shows the band intensities determined by Molecular Imaging Software version 4.0.

Dose-dependent inhibition of COX2 protein expression was observed under NS398 treatment. COX2-positive cell/total cell ratio was 80.5% in untreated control samples, whereas it decreased to 77.0% under 10 *μ*M, to 61.2% under 25 *μ*M NS398 treatment. Further elevation of the NS398 dose (100 *μ*M) caused a significant decrease in the positive cell ratio (53.1%). Strong granular and/or diffuse cytoplasmatic immunostaining was detected in COX2-positive cells (Figure 3A and B). Western blot results showed correlation to the immunocytochemistry findings (Figure 3C). More considerable reduction in COX2 protein expression was detected after 96 h of NS398 treatment at 50 and 100 *μ*M concentrations.

## Discussion

It is a known fact that COX2 inhibitor treatment leads to a significant reduction in the number of colorectal polyps in patients with familial adenomatous polyposis (Steinbach *et al*, 2000; Higuchi *et al*, 2003). Selective COX2 inhibitors also seem to be effective for prevention of sporadic adenomatous polyps, as they significantly reduced the occurrence of colorectal adenomas within 3 years after polypectomy (Arber *et al*, 2006). However, their use is associated with increased cardiovascular risk (Baron *et al*, 2006; Bertagnolli *et al*, 2006). The treatment of CRC patients with selective COX2 inhibitors should be less effective, because increased COX2 expression is present in the earlier phase of colorectal carcinogenesis (Eberhart *et al*, 1994; Yona and Arber, 2006), but the exact molecular biological reasons in the background of this phenomenon are not clarified yet. High-throughput screening technologies such as mRNA expression microarrays were applied to find other molecular targets of selective COX2 inhibitors besides COX2, in order to discover the mechanisms explaining their anti-cancer effect in prostate cancer (John-Aryankalayil *et al*, 2009; Sooriakumaran *et al*, 2009) and CRC (Zagani *et al*, 2009).

In this study, we analysed the effect of NS398 selective COX2 inhibitor on adenoma- and CRC-associated gene expression profiles in the HT29 colon adenocarcinoma cell line using the whole-genomic HGU133 Plus 2.0 microarray system. The global gene expression modulatory effect of NS398 was also examined to find other target molecules and pathways influenced by NS398 selective COX2 inhibitor treatment in epithelial cells.

We found that NS398 has a reverse effect on the expression of genes with altered expression in the colorectal adenoma–carcinoma sequence. NS398 more efficiently inverted the expression changes at the adenoma than in the carcinoma stage, demonstrating that it is an effective drug in CRC chemoprevention in the early phase of carcinogenesis.

We have previously identified CRC and adenoma-specific gene expression marker sets in biopsy samples for diagnostic classification. Although colorectal adenoma and adenocarcinoma are epithelial alterations, the cancer microenvironment and interaction between cancer and stromal cells have critical roles in tumour development and progression. That is why the origin of mRNA expression changes – identified in biopsy samples containing both epithelial and stromal tissue elements – was analysed using LCM epithelial samples before model selection. The HT29 colon adenocarcinoma cell line was selected after establishing the fact that most of the above-mentioned markers are epithelium derived. The other reason was that COX2 is decisively expressed in the adenomatous or tumourous epithelium, but there are several studies in which stromal COX2 expression is reported (Nakagawa *et al*, 2004; Soumaoro *et al*, 2004).

Our gene expression microarray results strengthen the previously published data by which the anti-cancer effect of the selective COX2 inhibitors is mainly due to their anti-proliferative and pro-apoptotic properties (Guardavaccaro *et al*, 2000; Hung *et al*, 2000; Brown *et al*, 2001; Li *et al*, 2001; Zhang and DuBois, 2001; Nishikawa *et al*, 2004; Chen *et al*, 2009; Sooriakumaran *et al*, 2009; Zagani *et al*, 2009). The cell proliferation inhibitory effect of NS398 could be detected in our microarray analysis by causing cell cycle arrest in the G1 phase, as described earlier (Hung *et al*, 2000). This can be mediated not only by p27KIP1 (Hung *et al*, 2000) but also by p18-INK4C (CDKN2C) and CIP2 (CDKN3) overexpression (the latter ones showed more than a 4.5-fold overexpression under NS398 treatment in our study). The p53-inducible gene BTG2 (we found to be 4.6-fold upregulated in HT29 cells after NS398 treatment) also contributes to the anti-proliferative activity of NS398 through its inhibition effect to G(1)–S transition by reduction of cyclin D1 levels (Guardavaccaro *et al*, 2000). The cell proliferation inhibitory effect of NS398 has also been proven in MTT assay.

The inhibition of COX2 by NS398 results in the accumulation of arachidonic acid in cancer cells and, therefore, would trigger apoptosis, but the mechanisms by which NSAIDs induce cancer cells to apoptosis can also be COX2 independent. In this study, a wide range of pro-apoptotic genes in different phases of apoptosis were found to be overexpressed under NS398 treatment including TRAIL death ligand (TNFSF10), SIVA1 death receptor in CD27-induced pathway and molecules involved in the execution phase of apopotosis such as the APAF1 apoptosome protein and CASP6 effector caspase. Death-associated kinase-3 (DAPK3) inducing morphological changes in apoptosis was also upregulated by NS398 in HT29 cells. In accordance with the findings of Li *et al* (2001), NS398-dependent apoptosis in colon cancer cells occurred through a cytochrome *c*-dependent pathway in our experiments. We found that the activation of the p53-dependent pathway can also trigger apoptotic processes via the cytochrome *c* pathway. Overexpression of tumour protein p53-inducible nuclear protein-1 and tumour protein p53-inducible protein-3 pro-apoptotic molecules indicates p53-dependent apoptosis. p73, which can transactivate p53-responsive genes causing cell cycle arrest and apoptosis, is also upregulated under NS398 COX2 inhibitor treatment. Celecoxib also caused overexpression of p73 tumour-suppressor gene in prostate cancer in a randomised controlled phase II pre-surgical trial (Sooriakumaran *et al*, 2009).

Although only few genes involved in angiogenesis showed significant mRNA expression changes, in accordance with observations of Abdelrahim and Safe (2005) and Huang *et al* (2005), we also detected the downregulation of VEGF, one of the most important angiogenic factors, besides the underexpression of others such as PTEN and IL18.

In summary, in this study, we analysed the effect of NS398 selective COX2 inhibitor treatment on colorectal adenoma- and CRC-associated gene expression alterations using whole-genomic mRNA expression microarrays and the HT29 colon adenocarcinoma cell line. Dose-dependent inhibition of COX2 protein expression was found to be associated with reversal gene expression pattern changes in the colorectal normal-adenoma but less in the normal-carcinoma pathway. Our findings can provide a molecular explanation with regard to the efficacy of selective COX2 inhibitors in CRC chemoprevention in the pre-cancerous adenoma phase. Furthermore, our results can give an insight into the global molecular background of selective COX2 inhibitor administration suggesting the involvement of p18-INK4C, CIP2 cyclin-dependent kinase inhibitors and p53-inducible BTG2 gene in NS398-dependent proliferation inhibition and TRAIL- and p53-mediated apoptotic pathways.

## Figures and Tables

**Table 1:** Classificatory genes identified by gene expression microarray analysis of colorectal biopsy samples

**Affymetrix ID**	**Gene symbol**	**Gene name**	**Function**
*Adenoma* vs *normal*			
212942_s_at	KIAA1199	KIAA1199	Sensory perception of sound
227475_at	FOXQ1	Forkhead box Q1	Transcription, regulation of transcription
207504_at	CA7	Carbonic anhydrase VII	One-carbon compound metabolic process
204719_at	ABCA8	ATP-binding cassette, subfamily A (ABC1), member 8	Transport
1552296_at	BEST4	Bestrophin 4	Ion transport
240157_at	—	—	—
205861_at	SPIB	Spi-B transcription factor	Transcription, regulation of transcription
207080_s_at	PYY	Peptide YY	Cell motility, cytoskeleton organisation and biogenesis, cell proliferation
237530_at	—	—	—
230204_at	HAPLN1	Hyaluronan and proteoglycan link protein 1	Cell adhesion
240389_at	TRPM6	Transient receptor potential cation channel, subfamily M, member 6	Protein amino acid phosphorylation, calcium ion transport
1558324_a_at	TMEM72	Transmembrane protein 72	—
214598_at	CLDN8	Claudin 8	Calcium-independent cell–cell adhesion
203256_at	CDH3	Cadherin 3, type 1, P-cadherin	Transcription, regulation of transcription
205523_at	HAPLN1	Hyaluronan and proteoglycan link protein 1	Cell adhesion
206422_at	GCG	Glucagon	Signal transduction, feeding behavior, cell proliferation
213921_at	SST	Somatostatin	Cell surface receptor linked signal transduction
224412_s_at	TRPM6	Transient receptor potential cation channel, subfamily M, member 6	Protein amino acid phosphorylation, calcium ion transport
203000_at	STMN2	Stathmin-like 2	Intracellular signalling cascade, neuron differentiation
202504_at	TRIM29	Tripartite motif-containing 29	Transcription from RNA polymerase II promoter
			
*CRC* vs *normal*			
202112_at	VWF	von Willebrand factor	Cell adhesion, blood coagulation, hemostasis
202859_x_at	IL8	Interleukin 8	Angiogenesis, cell motility, chemotaxis
209395_at	CHI3L1	Chitinase 3-like 1	Carbohydrate metabolic process, chitin catabolic process, transport
202917_s_at	S100A8	S100 calcium binding protein A8	Inflammatory response
218468_s_at	GREM1	Gremlin 1, cysteine knot superfamily, homolog	Multicellular organismal development, nervous system development
212531_at	LCN2	Lipocalin 2	Transport
204470_at	CXCL1	Chemokine (C-X-C motif) ligand 1	Chemotaxis, inflammatory response, cell proliferation
211981_at	COL4A1	Collagen, type IV, *α*1	Phosphate transport
213338_at	TMEM158	Transmembrane protein 158	—
211980_at	COL4A1	Collagen, type IV, *α*1	Phosphate transport
209752_at	REG1A	Regenerating islet-derived 1*α*	Positive regulation of cell proliferation
211959_at	IGFBP5	Insulin-like growth factor binding protein 5	Regulation of cell growth, signal transduction
225681_at	CTHRC1	Collagen triple helix repeat containing 1	Phosphate transport
218469_at	GREM1	Gremlin 1, cysteine knot superfamily, homolog	Multicellular organismal development, nervous system development
209087_x_at	MCAM	Melanoma cell adhesion molecule	Cell adhesion, anatomical structure morphogenesis
207504_at	CA7	Carbonic anhydrase VII	One-carbon compound metabolic process
205067_at	IL1B	Interleukin 1, *β*	Angiogenesis, fever, apoptosis, inflammatory response
212657_s_at	IL1RN	Interleukin 1 receptor antagonist	Inflammatory response, immune response
225664_at	COL12A1	Collagen, type XII, *α*1	Skeletal development, phosphate transport, cell adhesion
202311_s_at	COL1A1	Collagen, type I, *α*1	Skeletal development, ossification, phosphate transport
209774_x_at	CXCL2	Chemokine (C-X-C motif) ligand 2	Chemotaxis, inflammatory response
225842_at	PHLDA1	Pleckstrin homology-like domain, family A, member 1	Apoptosis, FasL biosynthetic process
219727_at	DUOX2	Dual oxidase 2	Electron transport, response to oxidative stress
211506_s_at	IL8	Interleukin 8	Angiogenesis, cell motility, chemotaxis, inflammatory response
209875_s_at	SPP1	Secreted phosphoprotein 1 (osteopontin)	Ossification, cell adhesion, negative regulation of bone mineralisation
205828_at	MMP3	Matrix metallopeptidase 3	Proteolysis, metabolic process
211340_s_at	MCAM	Melanoma cell adhesion molecule	Cell adhesion, anatomical structure morphogenesis
1552296_at	BEST4	Bestrophin 4	Ion transport
204677_at	CDH5	Cadherin 5, type 2, VE-cadherin	Cell adhesion
202404_s_at	COL1A2	Collagen, type I, *α*2	Skeletal development, phosphate transport
240157_at	—	—	—
39402_at	IL1B	Interleukin 1, *β*	Angiogenesis, fever, apoptosis, inflammatory response
206121_at	AMPD1	Adenosine monophosphate deaminase 1	Nucleotide metabolic process
201438_at	COL6A3	Collagen, type VI, *α*3	Phosphate transport, cell adhesion, muscle development
201195_s_at	SLC7A5	Solute carrier family 7, member 5	Amino acid metabolic process, transport
1558324_a_at	TMEM72	Transmembrane protein 72	—
210869_s_at	MCAM	Melanoma cell adhesion molecule	Cell adhesion, anatomical structure morphogenesis
220724_at	FLJ21511	Hypothetical protein FLJ21511	—

**Table 2:** Taqman validation of 12 selected discriminatory genes

**Taqman ID**	**Gene symbol**	**Gene name**	**Affymetrix ID**	**Compared sample groups**	**Fold change on microarrays**	**Fold change in RT–PCR 2** ^**(-**ΔΔ**CT)**^	***P*-value**
Hs00200350_m1	ABCA8	ATP-binding cassette, subfamily A (ABC1), member 8	204719_at	Adenoma *vs* normal	0.078	0.10	**0.00061**
Hs00214306_m1	TRPM6	Transient receptor potential cation channel, subfamily M, member 6	240389_at	Adenoma *vs* normal	0.086	0.04	**0.00006**
Hs00169795_m1	VWF	von Willebrand factor	202112_at	CRC *vs* normal	3.61	12.21	0.55142
Hs00174103_m1	IL8	Interleukin 8	202859_x_at	CRC *vs* normal	20.20	148.06	**0.00283**
Hs00194353_m1	LCN2	Lipocalin 2	212531_at	CRC *vs* normal	7.97	28.44	**0.00051**
Hs00236937_m1	CXCL1	Chemokine (C-X-C motif) ligand 1	204470_at	CRC *vs* normal	13.10	14.32	**0.01140**
Hs00266237_m1	COL4A1	Collagen, type IV, *α*1	211980_at	CRC *vs* normal	5.21	10.41	**0.02831**
Hs00174838_m1	MCAM	Melanoma cell adhesion molecule	209087_x_at	CRC *vs* normal	2.92	6.87	0.05209
Hs00277299_m1	IL1RN	Interleukin 1 receptor antagonist	212657_s_at	CRC *vs* normal	11.99	25.28	**0.00714**
Hs00236966_m1	CXCL2	Chemokine (C-X-C motif) ligand 2	209774_x_at	CRC *vs* normal	9.20	13.00	**0.00204**
Hs00204187_m1	DUOX2	Dual oxidase 2	219727_at	CRC *vs* normal	9.70	30.06	**0.00363**
Hs00167093_m1	SPP1	Secreted phosphoprotein 1 (osteopontin)	209875_s_at	CRC *vs* normal	8.92	12.55	0.07492

*P*-value represents the correlation to the microarray data. The significant different expression (*P*<0.05) is marked in bold.
