# Supplementary figures and images for: Reversal of gene expression changes in the colorectal normal-adenoma pathway by NS398 selective COX2 inhibitor
Source: Br J Cancer. 2010 Jan 19;102(4):765–73. doi: 10.1038/sj.bjc.6605515 (PMC2837560; doi:10.1038/sj.bjc.6605515)

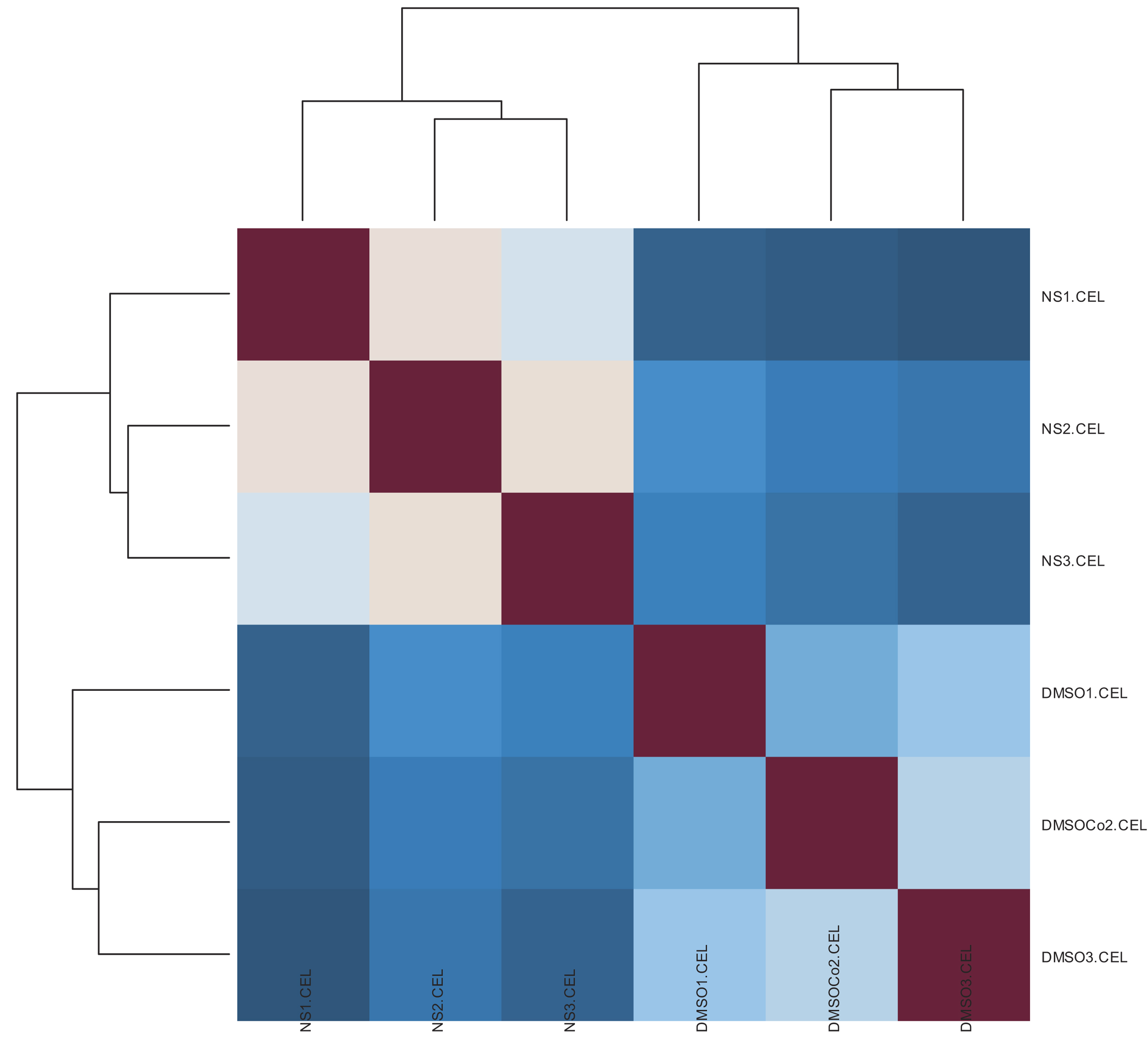

Supplement: Supplementary Figure 1 [file 6605515x1.tif]
