# Supplementary material for: Reversal of gene expression changes in the colorectal normal-adenoma pathway by NS398 selective COX2 inhibitor
Source: Br J Cancer. 2010 Jan 19;102(4):765–73. doi: 10.1038/sj.bjc.6605515 (PMC2837560; doi:10.1038/sj.bjc.6605515)
Supplement: Supplementary information [file 6605515x3.doc]

**Supplemental information**

**Supplemental Table 1**. **Differentially expressed genes between NS398-treated and untreated control HT29 colon adenocarcinoma cells**

Totally 1925 differentially expressed genes (1156 overexpressed and 769 downregulated) were identified between NS398-treated and untreated control group using SAM at a significance of p<0.05. The annotation and functional classification of discriminatory genes were performed using Affymetrix NetAffx system.

**Supplemental Figure 1. Euclidean distances of HT29 microarray experiments**

HT29 microarray experiments were performed in triplicate (from 3 samples treated with 100µM NS398 /NS1.CEL, NS2.CEL, NS3.CEL/ and from 3 untreated controls /DMSO1.CEL, DMSOCo2.CEL, DMSO3.CEL/). Euclidean method was applied to compute the distance between samples (for all approx. 54 000 variables). According to the Euclidean distances the two observed group containing triplicates could be clearly separated.
